# Self-dependent neural variability predicts recovery from depressive symptoms

**DOI:** 10.1093/scan/nsab050

**Published:** 2021-05-15

**Authors:** Leyi Fan, Qin Duan, Siyang Luo

**Affiliations:** Department of Psychology, Guangdong Key Laboratory of Social Cognitive Neuroscience and Mental Health, Guangdong Provincial Key Laboratory of Brain Function and Disease, Sun Yat-sen University, Guangzhou, Guangdong 510006, China; Department of Psychology, Guangdong Key Laboratory of Social Cognitive Neuroscience and Mental Health, Guangdong Provincial Key Laboratory of Brain Function and Disease, Sun Yat-sen University, Guangzhou, Guangdong 510006, China; Department of Psychology, Guangdong Key Laboratory of Social Cognitive Neuroscience and Mental Health, Guangdong Provincial Key Laboratory of Brain Function and Disease, Sun Yat-sen University, Guangzhou, Guangdong 510006, China

**Keywords:** self, fMRI, neural variability, recovery, depressive symptoms

## Abstract

Researchers have increasingly paid attention to the neural dynamics of depression. This study examined whether self-dependent neural variability predicts recovery from depressive symptoms. Sixty adults with depressive symptoms who were not officially diagnosed with major depressive disorder participated in this study. Participants completed functional magnetic resonance imaging (fMRI) scanning, including a resting-state and a self-reflection task. The fMRI data were used to estimate neural variability, which refers to the temporal variability in regional functional connectivity patterns. Participants then completed the Self-Construal Scale and the Beck Depression Inventory (BDI). The change in BDI scores over 3 months indicated the degree of recovery from depressive symptoms. Self-construal moderated the effects of general neural variability on predicting recovery from depressive symptoms. Interdependent individuals became less depressive with higher general neural variability, but the relationship was not significant in independent individuals. The differences in neural variability between self-related and other-related conditions also predicted recovery from depressive symptoms. The regions contributing to the prediction were mainly distributed in the default-mode network. Based on these results, the harmony between individuals’ neural dynamics and self-concept is important for recovery from depressive symptoms, which might be a foundation for individualized treatment and counseling.

## Introduction

Depression has affected over 264 million people worldwide (GBD 2017 Disease and Injury Incidence and Prevalence Collaborators, 2018). Based on studies examining the neurological mechanisms that underlie depression, various treatments were proposed to intervene in major depressive disorder (MDD), such as medications and cognitive therapies (Beck, 1979; van der Velden *et al.*, 2015; Trivedi *et al.*, 2016; Cohen and DeRubeis, 2018; Tozzi *et al.*, 2019). However, studies also found that only approximately one-quarter of individuals with temporary depressive symptoms develop chronic depression, whereas others recover without treatment (DeRubeis *et al.*, 2008; Houle *et al.*, 2013; van Grieken *et al.*, 2015). To date, the process of recovering from depressive symptoms without treatment has rarely been studied.

Based on accumulating evidence, self-related processing is crucial to depression. MDD is associated with an increased self-focus by which one engages in self-referential processing (Derry and Kuiper, 1981; Watkins and Teasdale, 2004; Grimm *et al.*, 2009; Nejad *et al.*, 2013). Negative self-referential bias predicts a deterioration of depressive symptoms (Disner *et al.*, 2017; LeMoult *et al.*, 2017). Self-related processing was found to be associated with specific neural activity in midline regions of the brain, such as the default-mode network (DMN), the anterior cingulate cortex (ACC) and the medial prefrontal cortex (mPFC) (Northoff and Bermpohl, 2004; Northoff *et al.*, 2006; Qin *et al.*, 2013, 2016). Consistent with behavioral findings, individuals with MDD show abnormalities in the DMN originating from global sources, both within- and intra-DMN sources (Sheline *et al.*, 2009; Scalabrini *et al.*, 2020). Self- *vs* non-self-judgment elicits neural activity in the dorsal medial frontal gyrus (MFG) and the dorsolateral prefrontal cortex (DLPFC) and enhances functional connectivity between them in patients with MDD (Lemogne *et al.*, 2009). Abnormally increased self-focus in patients with MDD is related to neural activity in subcortical–cortical midline structures (Grimm *et al.*, 2009). Both behavioral and neural evidence show that self-related processing plays an important role in depressive symptoms.

Moreover, social relationships and cultural circumstances are associated with depression (Gore *et al.*, 1993; Kleinman and Good, 2004; Teo *et al.*, 2013; Kirmayer *et al.*, 2017). Self-construal refers to the self-definition and interpretation of individuals (Markus and Kitayama, 1991; Singelis, 1994; Wagar and Cohen, 2003; Knyazev *et al.*, 2020), describing the self from a sociocultural perspective. Interdependent self-construal, which is dominant in Eastern collectivistic cultures, defines the self in the light of social contexts and others. Independent self-construal, which is dominant in Western individualistic cultures, defines the self as an autonomous and bounded entity (Markus and Kitayama, 1991). Studies found that self-construal is related to depression. Bae (1999) reported a positive correlation between interdependent self-construal and depressive symptoms and a negative correlation between independent self-construal and depressive symptoms. In addition, Lam (2005) found that interdependent self-construal indirectly affects depression by increasing family cohesion, which enhances self-esteem among Vietnamese-American adolescents. Therefore, self-construal may modulate the process of depression.

Neural studies have found that brain networks, including the DMN and the salience and emotion network (SEN), are closely related to depression (Yeo *et al.*, 2011; Wang *et al.*, 2012; Kaiser *et al.*, 2015; Jacobs *et al.*, 2016). Neural dynamics have recently received increasing attention in psychiatric and neurological disorders. The temporal variability in regions of the DMN differs between patients with distinct mental disorders (Zhang *et al.*, 2016). Patients with MDD present higher temporal variability in the right inferior frontal gyrus, the left inferior occipital gyrus, the bilateral fusiform gyri and the left Heschl’s gyrus than healthy controls (Hou *et al.*, 2018). Researchers have also suggested that temporal variability might be a promising indicator for individualized therapy of MDD (Hou *et al.*, 2018). Based on these findings, researchers speculated that temporal variability might be an indicator of brain flexibility and adaptability or a predictor of neural rehabilitation (Zhang *et al.*, 2016), which in turn might affect recovery from depression.

This study aimed to investigate the neural dynamics of recovery from depressive symptoms. We hypothesized that self-dependent neural variability predicts recovery from depressive symptoms. Brain regions associated with self-related processing, including the DMN, contribute to the prediction.

## Materials and methods

### Participants

Two hundred thirty-nine individuals participated in depressive symptom screening. These participants were asked to complete the Beck Depression Inventory-II (BDI-II; Beck *et al.*, 1996). The BDI included 21 sets of statements with four statements in each set. Sixty individuals (42 males and 18 females; age = 20.11 ± 2.33 years) participated in the functional magnetic resonance imaging (fMRI) studies. Among the 60 individuals, the 28 participants with a BDI score higher than 14 (range: 14–28, mean ± s.d. = 19.21 ± 2.19) were assigned to the high-depression group and the other 32 participants with a BDI score lower than 4 (range: 0–4, mean ± s.d. = 2.25 ± 1.44) were assigned to the low-depression group. The participants completed the BDI again 3 months after the first screen. Recovery from depressive symptoms was assessed by subtracting the BDI score recorded at the second screen from the BDI score reported at the first screen. Higher scores indicated that the participants had become less depressive. None of the participants took psychotropic medicine or treatment between the two screens. Written informed consent was obtained from all participants before starting the experiments. All studies were approved by the ethics committee of the Department of Psychology at Sun Yat-sen University.

### Procedure and stimuli

**Fig. 1.**
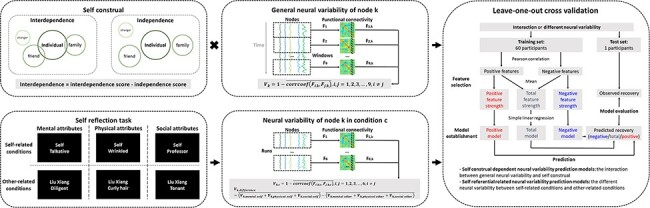
Flowchart of the prediction analysis. The neural variability of a node was defined as the temporal variability of functional connectivity in the node. In the self-construal-dependent neural variability predictive models, self-dependent neural variability was defined as the interaction between general neural variability and interdependence. In the self-referential-related neural variability predictive models, self-dependent neural variability was defined as the difference in neural variability between self-related conditions and other-related conditions during the self-reflection task. The leave-one-out cross-validation method was used to study whether self-dependent neural variability predicted recovery from depressive symptoms.

During fMRI scanning, the participants completed a 5-min resting-state and a self-reflection task (Figure 1). During the task, the participants were asked to judge whether a particular item described themselves (self-related conditions) or a public figure (other-related conditions; i.e. Liu Xiang, a famous athlete). The items were divided into three categories: mental attributes (i.e. personality characteristics), physical attributes (i.e. physical appearances) and social attributes (i.e. social identities). Each category contained 48 items. A font judgment (bold *vs* light) was used as the control condition. Six scans with seven blocks in each scan were performed. Each block presented a type of judgment. The blocks were presented in a random order and with a 10-s interval. Each item was presented for 2 s and followed by a 1-s fixation.

After scanning, participants completed the Self-Construal Scale (Singelis, 1994). The Self-Construal Scale was divided into two dimensions: the interdependent subscale and the independent subscale. Each subscale included 12 items that were rated on a 7-point Likert scale (1 = strongly disagree and 7 = strongly agree). Interdependence was assessed by subtracting the mean score for the independent subscale from the mean score for the interdependent subscale (Figure 1). Higher scores indicated greater interdependence on social contexts and others.

### Imaging acquisition and preprocessing

We used a GE Signa MR750 3.0T scanner with a standard head coil to acquire the fMRI data. The data were acquired using T2-weighted, gradient-echo, echo-planar imaging (EPI) sequences with the following parameters: repetition time (TR) = 2000 ms, echo time (TE) = 30 ms, flip angle (FA) = 90°, field of view (FOV) = 240 × 240 mm, matrix = 64 × 64 × 32 and spatial resolution = 3.75 × 3.75 × 5 mm^3^. During resting-state scanning, the participants were asked to keep their eyes open.

A standard preprocessing procedure was performed on the fMRI data using the Data Processing Assistant for Resting-State fMRI (DPARSF) toolbox (Yan and Zang, 2010). The data from the first five volumes were removed. The remaining data underwent slice timing and realignment to correct the time delay of scans and head motion. None of the participants was excluded during realignment because their maximum head motion was all within the criterion of 3.0 mm and 3.0°. The corrected data were registered to the Montreal Neurological Institute (MNI) space with the EPI template. The normalized data were Gaussian smoothed [full-width at half-maximum (FWHM) = 4 mm], detrended, and bandpass filtered (0.01–0.08 Hz). Nuisance covariates were removed by multiple regressions, including six rigid-body head motion parameters and the mean time courses of the white matter and cerebrospinal fluid. In addition, we used different nuisance covariate removal strategies (removal of the global signal *vs* no removal) to test the robustness of the prediction effects across nuisance covariate removal strategies (see supplementary results).

A 264-node atlas (Power *et al.*, 2011) was used to define nodes and networks. The atlas included the cerebral cortex, subcortical structures and cerebellum. The nodes were 264 spheres (diameter = 10 mm) divided into 14 networks (see supplementary methods).

### Estimation of neural variability

We used the method proposed by Zhang *et al.* (2016) to estimate neural variability. The neural variability of each node was defined as the temporal variability of functional connectivity in the node.

#### Estimation of general neural variability.

We used the fMRI data collected during the resting state to estimate general neural variability. Time series were extracted and split into nine non-overlapping windows with a length of 30 s. In space, we calculated the functional connectivity matrix within each window using Pearson’s correlation analysis. Each row (or each column) of the matrix represented the functional connectivity between a specific node and the remaining nodes across the whole brain. For each node, we compared the functional connectivity matrices across different windows of time using Pearson’s correlation analysis. General neural variability of the node was calculated by subtracting the mean of correlation coefficients from one. The following formula was used:

$$\begin{equation*}{V_k} = 1 - \overline {corrcoef\left( {{F_{i.k,}}{F_{j.k}}} \right)} ,i,j = 1,2,3, \ldots ,9,i \ne j,\end{equation*}$$

where *k* indicated the node and *i* and *j* indicated windows.

#### Estimation of the difference in neural variability between the self-related conditions and other-related conditions.

We used the fMRI data collected during the self-reflection task to estimate the difference in neural variability between the self-related conditions and other-related conditions. The neural variability of each condition was calculated by comparing regional functional connectivity matrices across different scans under the same condition. The procedure was similar to the estimation of general neural variability. The difference in neural variability was calculated by subtracting the mean neural variability in the three other-related conditions from the mean neural variability in the three self-related conditions.

### Prediction analysis

We used the leave-one-out cross-validation method to study whether self-dependent neural variability predicted recovery from depressive symptoms in a novel individual (Figure 1). In each iteration, one participant was excluded as the test set and the remaining participants were the training set. The training set established the predictive models, and the test set evaluated the predictive models. Because each of the 60 participants was excluded once, 60 iterations were performed.

#### Self-construal-dependent neural variability predictive models.

Before the prediction analysis, the dot product of normalized general neural variability and normalized interdependence was defined as the interaction between general neural variability and interdependence. We used the interaction between general neural variability and interdependence to study whether self-construal moderated the effects of general neural variability on predicting the recovery from depressive symptoms in a novel individual.

In the feature-selection stage, based on the training set, we performed Pearson’s correlation analysis between the interactions in each node and recovery scores (feature-selection threshold = 0.05). The nodes whose interactions were significantly positively correlated with recovery were selected as positive features, and the significantly negatively correlated nodes were negative features. In addition, we used different feature-selection thresholds (thresholds = 0.05 and 0.01) to test the robustness of the prediction effects across feature-selection thresholds (see supplementary methods and results).

In the model establishment stage, positive features or the opposite number of negative features were averaged, resulting in the positive feature interaction strength or the negative feature interaction strength, respectively, and they were averaged together, resulting in the total feature interaction strength. Simple linear regression analyses were conducted to construct the relationships between the three feature interaction strengths and recovery, resulting in three models: the total model, positive model and negative model.

In the prediction stage, the same features as the training set were extracted from test set, and the three interaction strengths were substituted in the corresponding model, resulting in the predicted recovery for the participant in each of the three models.

In the model evaluation stage, the correlation coefficient between the predicted recovery calculated using the model and the observed recovery assessed using the scale was defined as the predictive power of the model. Only significant positive predictive power indicated that the prediction was successful. The nodes that were selected as features in more than 95% of the iterations were regarded as important nodes. We performed permutation tests to further confirm the significance of the predictions (see supplementary methods).

#### Self-referential-related neural variability predictive models.

The procedure was similar to the prediction analyses of the interaction between general neural variability and interdependence except that the interaction was replaced with the differences in neural variability between self-related and other-related conditions. Moreover, for the domain-specific prediction effect, we used the differential neural variability between self-related conditions and other-related conditions in which participants made judgments about mental attributes, physical attributes or social attributes to perform the prediction analyses (see supplementary methods).

## Results

### Self-construal-dependent neural variability predictive models

**Fig. 2.**
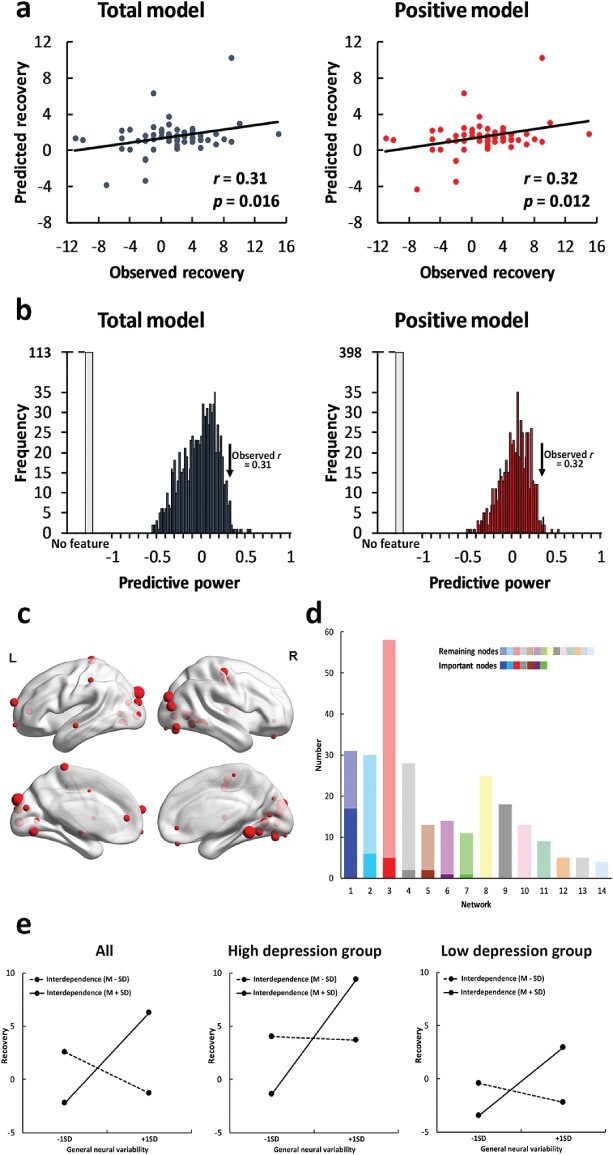
Results of the self-construal-dependent neural variability predictive models. (a) The predictive power (correlation between the predicted recovery and the observed recovery) of the total model (left panel, dark gray) and positive model (right panel, red). (b) The results of permutation tests in the total model (left panel, dark gray) and positive model (right panel, red). The black arrow indicates the observed predictive power in the two models. The light gray bar indicates the permutations in which no feature was selected in at least one iteration. (c) The locations of the important nodes (nodes that were selected as features in more than 95% of the iterations). (d) The distribution of the important nodes in the visual network (blue), sensory–somatomotor network (cyan), DMN (red), uncertain network (striated), subcortical network (brown), cingulo-opercular task control network (purple), dorsal attention network (green), frontoparietal task control network (yellow), salience network (black), auditory network (pink), hand ventral attention network (teal), mouth sensory–somatomotor network (orange), memory retrieval network (gray) and cerebellar network (pale blue). The dark colors indicate the important nodes, and the light colors indicate the remaining nodes. The networks were ranked by the number of the important nodes. (e) The simple effects on all participants (left panel), high-depression group (middle panel) and low-depression group (right panel).

The interaction between general neural variability and interdependence successfully predicted recovery from depressive symptoms in the total model (*r* = 0.31, *P* = 0.016, Figure 2A) and in the positive model (*r* = 0.32, *P* = 0.012). In the negative model, no feature was selected in at least one iteration. The prediction effects were robust across nuisance covariate removal strategies (no removal of the global signal *vs* removal) and feature selection thresholds (thresholds = 0.05, 0.01) (see supplementary results). However, general neural variability itself failed to predict recovery from depressive symptoms in the three models (total model: *r* = 0.13, *P* = 0.323; positive model: *r* = 0.23, *P* = 0.070; negative model: *r* = −0.23, *P* = 0.081). The results indicated that self-construal moderated the effects of general neural variability on predicting recovery from depressive symptoms in individuals. Permutation tests further confirmed that the observed predictive power significantly differed from the predictive power in the null distribution (total model: *P* = 0.015; positive model: *P* = 0.009, Figure 2B). Thirty-four important nodes were identified, all of which were positive features (Figure 2C). The important nodes were mainly distributed in the visual network (*n* = 17, Figure 2D), sensory–somatomotor network (*n* = 6) and DMN (*n* = 5).

We conducted a simple effects analysis with the mean general neural variability of the important nodes to examine the relationship between general neural variability and recovery from depressive symptoms in individuals with high interdependence (M + s.d.) and in individuals with low interdependence (M − s.d.). General neural variability was positively correlated with recovery from depressive symptoms (*b* = 4.24, SE = 1.35, *P* = 0.003, Figure 2E) in interdependent individuals, but was not significant in independent individuals (*b* = −1.95, SE = 1.03, *P* = 0.063). The simple effects were consistent on the high-depression group (interdependent individuals: *b* = 5.58, SE = 2.13, *P* = 0.015; independent individuals: *b* = 0.12, SE = 1.41, *P* = 0.935) and the low-depression group (interdependent individuals: *b* = 3.10, SE = 1.34, *P* = 0.028; independent individuals: *b* = −1.08, SE = 1.48, *P* = 0.471). Based on the results, interdependent individuals became less depressive if they had higher general neural variability.

### Self-referential-related neural variability predictive models

**Fig. 3.**
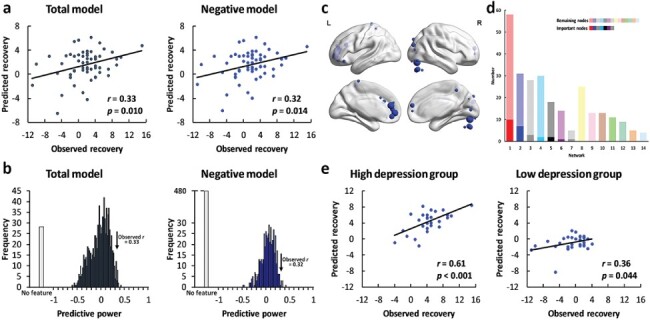
Results of the self-referential-related neural variability predictive models. (a) The predictive power of the total model (left panel, dark gray) and negative model (right panel, blue). (b) The results of permutation tests in the total model (left panel, dark gray) and negative model (right panel, blue). The black arrow indicates the observed predictive power in the two models. The light gray bar indicates the permutations in which no feature was selected in at least one iteration. (c) The locations of the important nodes. (d) The distribution of the important nodes. The colors of the networks are the same as those in Figure 2. The dark colors indicate the important nodes, and the light colors indicate the remaining nodes. The networks were ranked by the number of the important nodes. (e) The predictive power in the high-depression group (left panel) and low-depression group (right panel).

The differences in neural variability between the self-related conditions and other-related conditions during the self-reflection task successfully predicted recovery from depressive symptoms in the total model (*r* = 0.33, *P* = 0.010, Figure 3A) and in the negative model (*r* = 0.32, *P* = 0.014). In the positive model, no feature was selected in at least one iteration. The prediction effects were robust across feature selection thresholds (thresholds = 0.05, 0.01; see supplementary results). However, the differences in neural variability between the self-related conditions and font condition (total model: *r* = 0.15, *P* = 0.255; negative model: *r* = 0.15, *P* = 0.255; positive model: no feature) or the differences in neural variability between the other-related conditions and font condition (total model: *r* = −0.19, *P* = 0.145; negative model: *r* = −0.15, *P* = 0.244; positive model: *r* = 0.08, *P* = 0.525) failed to predict recovery from depressive symptoms. Thus, the difference between self-related neural variability and other-related neural variability predicted recovery from depressive symptoms in individuals. Permutation tests further confirmed that the observed predictive power significantly differed from the predictive power in the null distribution (total model: *P* = 0.012; negative model: *P* = 0.004, Figure 3B). Twenty-seven important nodes were identified, among which 26 nodes were negative features and one node was a positive feature (Figure 3C). The important nodes were mainly distributed in the DMN (*n* = 10, Figure 3D) and visual network (*n* = 7).

**Fig. 4.**
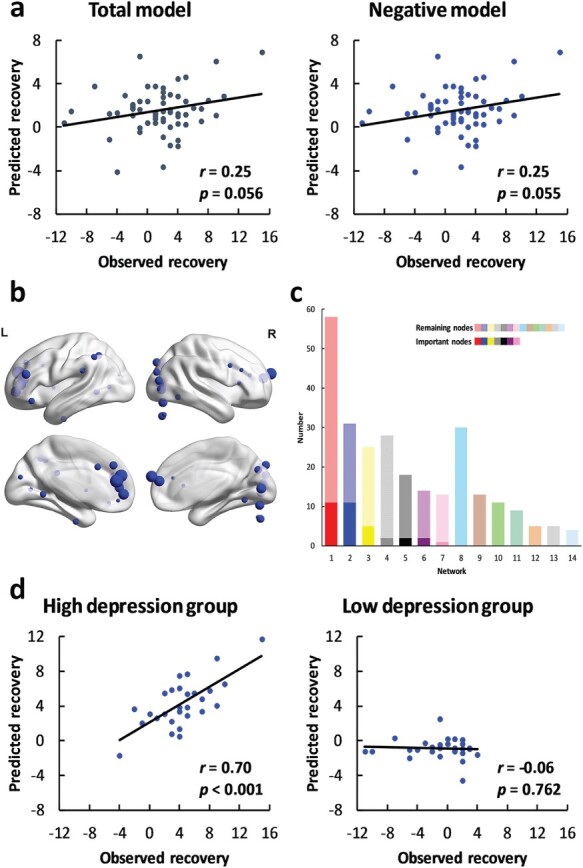
Results of the self-referential-related neural variability predictive models in the mental condition. (a) The predictive power of the total model (left panel, dark gray) and negative model (right panel, blue). (b) The locations of the important nodes. (c) The distribution of the important nodes. The colors of the networks are the same as those in Figure 2. The dark colors indicate the important nodes, and the light colors indicate the remaining nodes. The networks were ranked by the number of the important nodes. (d) The predictive power in the high-depression group (left panel) and low-depression group (right panel).

Moreover, the difference in neural variability between the self-related conditions and other-related conditions in which participants made judgments about mental attributes marginally significantly predicted recovery from depressive symptoms in the total model (*r* = 0.25, *P* = 0.056, Figure 4A) and in the negative model (*r* = 0.25, *P* = 0.055). The prediction effects were robust across feature selection thresholds (thresholds = 0.05, 0.01; see supplementary results). Thirty-four important nodes were identified, all of which were negative features (Figure 4B). The important nodes were mainly distributed in the DMN (*n* = 11, Figure 4C) and visual network (*n* = 11). However, the differences in neural variability in the physical condition or in the social condition failed to predict recovery from depressive symptoms (physical attribute—total model: *r* = −0.01, *P* = 0.967; negative model: *r* = −0.01, *P* = 0.923; positive model: no feature. Social attribute—total model: *r* = −0.17, *P* = 0.205; negative model: *r* = −0.03, *P* = 0.834; positive model: *r* = −0.10, *P* = 0.431). The results indicated the importance of the mental aspect of the self.

We examined the robustness of the predictive models in the high-depression group and in the low-depression group using the important nodes as features. The differences in neural variability within the important nodes successfully predicted recovery from depressive symptoms in both the high-depression group (*r* = 0.61, *P* < 0.001, Figure 3E) and the low-depression group (*r* = 0.36, *P* = 0.044). In the mental condition, the prediction effect was significant in the high-depression group (*r* = 0.70, *P* < 0.001, Figure 4D), but not in the low-depression group (*r* = −0.06, *P* = 0.762).

## Discussion

The present study provided the first evidence for the relationship between neural dynamics and mental dynamics (recovery from depression). Interdependent individuals with higher general neural variability in the resting state recovered better from depression, while independent individuals with higher neural variability showed no recovery. Interdependent individuals interpret their thoughts, feelings and actions in association with connectedness and integration with the external social environments, and independent individuals comprehend their thoughts, feelings and actions concerning separateness and uniqueness from within rather than reference to others (Markus and Kitayama, 1991). Interdependent individuals must consider and integrate more external information to link to various people and maintain this interconnectedness (Yeh and Hwang, 2000). In the process of adapting to various external environmental demands, the brain continues to modify its function and structure by strengthening, weakening, pruning or adding synaptic connections and by promoting neurogenesis (Pascual-Leone *et al.*, 2005, 2011). This finding is consistent with our results mentioned above; neural variability may be an indicator of brain flexibility and adaptability (Zhang *et al.*, 2016). Taken together, these results suggest that the harmony between self-construal and neural variability plays an important role in individuals’ mental health.

Moreover, the differences in neural variability between self-related conditions and other-related conditions successfully predicted recovery from depressive symptoms. A greater difference in neural variability between self-related conditions and other-related conditions was related to better recovery from depression symptoms. Analogously, previous research has emphasized the important role of self-related processing in depression, which has previously been reported in several studies (Sheline *et al.*, 2009; Disner *et al.*, 2017). This result is also consistent with a previous study showing that neural variability in the left pallidum is positively correlated with recovery from depressive symptoms (Hou *et al.*, 2018). Neural variability in the left pallidum predicts implicit self-esteem (Izuma *et al.*, 2018), which is closely related to depression (Battle, 1978; Orth *et al.*, 2016; Rieger *et al.*, 2016).

Furthermore, the DMN contributed to the prediction. The important nodes in our task-based predictive models were mainly distributed in the DMN. The DMN is involved in various self-related processes, including self-referential thoughts (Gusnard *et al.*, 2001), internal thought (Andrews-Hanna, 2012), autobiographical memory and theory of mind (Spreng and Grady, 2010). Furthermore, several studies found that the DMN is involved in self-related processing in individuals with depression (Lemogne *et al.*, 2009; Sheline *et al.*, 2009; Grimm *et al.*, 2011). For instance, negative blood-oxygen-level-dependent (BOLD) responses decreased in the DMN when patients with MDD made judgments on self-relatedness (Grimm *et al.*, 2011). The present study extended these findings from a dynamic perspective by showing that self-related neural variability in the DMN contributes to recovery from depression.

Moreover, the prediction effects of self-related neural variability were significant only when participants made judgments about mental attributes. Thus, different aspects of self may not be related to depression in a similar manner. Patients with MDD exhibit increased neural activity in the DLPFC and dorsal MFG and functional connectivity between the DLPFC, MFG and dorsal anterior cingulate cortex (dACC) when they made judgments on personality characteristics describing themselves rather than general desirable characteristics (Lemogne *et al.*, 2009). Meanwhile, these brain regions have been shown to correspond to important parts of the neural mechanisms underlying depression (Sheline *et al.*, 2009; Kaiser *et al.*, 2015; Jacobs *et al.*, 2016). The neural variability in the mental aspect of self-related processing appears to be particularly important for recovery from depression.

We used the leave-one-out cross-validation method to study whether self-dependent neural variability predicted recovery from depressive symptoms. Leave-one-out cross-validation was evidenced to provide an almost unbiased estimate of the probability of test error (Cawley and Talbot, 2003) from limited data. The method enabled us to test whether the predictive models established using the training data were generalizable to a novel observation. The results supported the generalization of the prediction effects.

One limitation of our study was that all the participants were Chinese. As previously shown, interdependent self-construal is adaptive and dominant in Eastern Asian culture, but independent self-construal is adaptive and dominant in Western culture (Singelis, 1994). Danish participants show greater activation in the mPFC when making judgments of self *vs* a public figure than Chinese participants, regardless of the attribute dimensions for the judgments, while greater activity in the temporoparietal junction (TPJ) was induced in Chinese participants than in Danish participants when making self-judgments of social attributes (Ma *et al.*, 2014). Researchers have suggested that people in different social and cultural contexts might adopt different self-reflection strategies by changing the neural activity of the mPFC and TPJ in social brain networks (Ma *et al.*, 2014). The present study illustrated that only interdependent Chinese participants became less depressive with higher general neural variability. Based on previous research, this relationship might be different in Western culture. Further studies should explore whether self-dependent neural variability remains a predictor of recovery from depressive symptoms in samples from a Western culture where independent self-construal is dominant. Moreover, future research should also examine the neural dynamics of the process from depressive symptom onset to the diagnosis of depression to test whether self-dependent neural variability also adequately predicts the development of depression.

## Conclusions

Taken together, our findings suggest that self-dependent neural variability predicts recovery from depressive symptoms in individuals. The effects of general neural variability on predicting recovery from depressive symptoms may be moderated by self-construal. Interdependent individuals with higher general neural variability in the resting state recover better from depressive symptoms. The difference between self-related neural variability and other-related neural variability predicted the recovery from depressive symptoms, especially in the mental domain. Our results provide the inspiration to understand and improve individuals’ mental health, which might be a foundation for individualized treatment and counseling in the future.

## Supplementary Material

nsab050_Supp

## Data Availability

The de-identified data that support the main findings of this study are available from the corresponding author upon reasonable request.

## Code availability

The custom computer code that was used in the main analysis of this study is available from the corresponding author.

## References

[R1] Andrews-Hanna J.R. (2012). The brain’s default network and its adaptive role in internal mentation. *The Neuroscientist*, 18, 251–70. 21677128 10.1177/1073858411403316 PMC3553600

[R2] Bae K. (1999). Multidimensional measures of acculturation and ethnic attachment as predictors of depressive symptoms in two populations of Korean-Americans. PhD Thesis. University of Michigan.

[R3] Battle J. (1978). Relationship between self-esteem and depression. *Psychological Reports*, 42, 745–6. 674498 10.2466/pr0.1978.42.3.745

[R4] Beck A.T. (1979). *Cognitive Therapy of Depression*. New York: Guilford Press.

[R5] Beck A.T., Steer R.A., Brown G.K. (1996). *Manual for the Beck Depression Inventory-II*. San Antonio: The Psychological Corporation.

[R6] Cawley G.C., Talbot N.L. (2003). Efficient leave-one-out cross-validation of kernel fisher discriminant classifiers. *Pattern Recognition*, 36(11), 2585–92.

[R7] Cohen Z.D., DeRubeis R.J. (2018). Treatment selection in depression. *Annual Review of Clinical Psychology*, 14, 209–36. 10.1146/annurev-clinpsy-050817-084746 29494258

[R8] Derry P.A., Kuiper N.A. (1981). Schematic processing and self-reference in clinical depression. *Journal of Abnormal Psychology*, 90(4), 286. 10.1037//0021-843x.90.4.286 7264058

[R9] DeRubeis R.J., Siegle G.J., Hollon S.D. (2008). Cognitive therapy versus medication for depression: treatment outcomes and neural mechanisms. *Nature Reviews Neuroscience*, 9, 788–96. 18784657 10.1038/nrn2345 PMC2748674

[R10] Disner S.G., Shumake J.D., Beevers C.G. (2017). Self-referential schemas and attentional bias predict severity and naturalistic course of depression symptoms. *Cognition and Emotion*, 31, 632–44. 26901406 10.1080/02699931.2016.1146123

[R11] GBD 2017 Disease and Injury Incidence and Prevalence Collaborators. (2018). Global, regional, and national incidence, prevalence, and years lived with disability for 354 diseases and injuries for 195 countries and territories, 1990–2017: a systematic analysis for the Global Burden of Disease Study 2017. *The Lancet*, 392, 1789–858. 10.1016/S0140-6736(18)32279-7 PMC6227754 30496104

[R12] Gore S., Aseltine Jr R.H., Colten M.E. (1993). Gender, social-relationship involvement, and depression. *Journal of Research on Adolescence*, 3(2), 101–25.

[R13] Grimm S., Ernst J., Boesiger P., et al. (2009). Increased self-focus in major depressive disorder is related to neural abnormalities in subcortical-cortical midline structures. *Human Brain Mapping*, 30(8), 2617–27. 19117277 10.1002/hbm.20693 PMC6870821

[R14] Grimm S., Ernst J., Boesiger P., Schuepbach D., Boeker H., Northoff G. (2011). Reduced negative BOLD responses in the default-mode network and increased self-focus in depression. *The World Journal of Biological Psychiatry*, 12, 627–37. 21247256 10.3109/15622975.2010.545145

[R15] Gusnard D.A., Akbudak E., Shulman G.L., Raichle M.E. (2001). Medial prefrontal cortex and self-referential mental activity: relation to a default mode of brain function. *Proceedings of the National Academy of Sciences of the United States of America*, 98, 4259–64. 11259662 10.1073/pnas.071043098 PMC31213

[R16] Hou Z., Kong Y., He X., Yin Y., Zhang Y., Yuan Y. (2018). Increased temporal variability of striatum region facilitating the early antidepressant response in patients with major depressive disorder. *Progress in Neuro-psychopharmacology and Biological Psychiatry*, 85, 39–45. 29608926 10.1016/j.pnpbp.2018.03.026

[R17] Houle J., Gascon-Depatie M., Bélanger-Dumontier G., Cardinal C. (2013). Depression self-management support: a systematic review. *Patient Education and Counseling*, 91, 271–9. 23414831 10.1016/j.pec.2013.01.012

[R18] Izuma K., Kennedy K., Fitzjohn A., Sedikides C., Shibata K. (2018). Neural activity in the reward-related brain regions predicts implicit self-esteem: a novel validity test of psychological measures using neuroimaging. *Journal of Personality and Social Psychology*, 114, 343–57. 29461079 10.1037/pspa0000114

[R19] Jacobs R.H., Barba A., Gowins J.R., et al. (2016). Decoupling of the amygdala to other salience network regions in adolescent-onset recurrent major depressive disorder. *Psychological Medicine*, 46, 1055–67. 26784396 10.1017/S0033291715002615 PMC4810773

[R20] Kaiser R.H., Andrews-Hanna J.R., Wager T.D., Pizzagalli D.A. (2015). Large-scale network dysfunction in major depressive disorder: a meta-analysis of resting-state functional connectivity. *JAMA Psychiatry*, 72, 603–11. 25785575 10.1001/jamapsychiatry.2015.0071 PMC4456260

[R21] Kirmayer L.J., Gomez-Carrillo A., Veissière S. (2017). Culture and depression in global mental health: an ecosocial approach to the phenomenology of psychiatric disorders. *Social Science and Medicine*, 183, 163–8. 28478959 10.1016/j.socscimed.2017.04.034

[R22] Kleinman A., Good B. (2004). Culture and depression. *New England Journal of Medicine*, 351, 951–2. 10.1056/NEJMp048078 15342799

[R23] Knyazev G.G., Savostyanov A.N., Bocharov A.V., Levin E.A., Rudych P.D. (2020). The default mode network in self-and other-referential processing: effect of cultural values. *Culture and Brain*, 1–17.

[R24] Lam B.T. (2005). Self-construal and depression among Vietnamese-American adolescents. *International Journal of Intercultural Relations*, 29(2), 239–50.

[R25] Lemogne C., Le Bastard G., Mayberg H., et al. (2009). In search of the depressive self: extended medial prefrontal network during self-referential processing in major depression. *Social Cognitive and Affective Neuroscience*, 4, 305–12. 19307251 10.1093/scan/nsp008 PMC2728628

[R26] LeMoult J., Kircanski K., Prasad G., Gotlib I.H. (2017). Negative self-referential processing predicts the recurrence of major depressive episodes. *Clinical Psychological Science*, 5, 174–81. 28286705 10.1177/2167702616654898 PMC5341388

[R27] Ma Y., Bang D., Wang C., et al. (2014). Sociocultural patterning of neural activity during self-reflection. *Social Cognitive and Affective Neuroscience*, 9, 73–80. 22956678 10.1093/scan/nss103 PMC3871729

[R28] Markus H.R., Kitayama S. (1991). Culture and the self: implications for cognition, emotion, and motivation. *Psychological Review*, 98, 224–53.

[R29] Nejad A.B., Fossati P., Lemogne C. (2013). Self-referential processing, rumination, and cortical midline structures in major depression. *Frontiers in Human Neuroscience*, 7, 666. 10.3389/fnhum.2013.00666 PMC3794427 24124416

[R30] Northoff G., Heinzel A., De Greck M., Bermpohl F., Dobrowolny H., Panksepp J. (2006). Self-referential processing in our brain—a meta-analysis of imaging studies on the self. *NeuroImage*, 31(1), 440–57. 16466680 10.1016/j.neuroimage.2005.12.002

[R31] Northoff G., Bermpohl F. (2004). Cortical midline structures and the self. *Trends in Cognitive Sciences*, 8(3), 102–7. 15301749 10.1016/j.tics.2004.01.004

[R32] Orth U., Robins R.W., Meier L.L., Conger R.D. (2016). Refining the vulnerability model of low self-esteem and depression: disentangling the effects of genuine self-esteem and narcissism. *Journal of Personality and Social Psychology*, 110, 133–49. 25915133 10.1037/pspp0000038

[R33] Pascual-Leone A., Amedi A., Fregni F., Merabet L.B. (2005). The plastic human brain cortex. *Annual Review of Neuroscience*, 28, 377–401. 10.1146/annurev.neuro.27.070203.144216 16022601

[R34] Pascual-Leone A., Freitas C., Oberman L., et al. (2011). Characterizing brain cortical plasticity and network dynamics across the age-span in health and disease with TMS-EEG and TMS-fMRI. *Brain Topography*, 24, 302–15. 21842407 10.1007/s10548-011-0196-8 PMC3374641

[R35] Power J.D., Cohen A.L., Nelson S.M., et al. (2011). Functional network organization of the human brain. *Neuron*, 72, 665–78. 22099467 10.1016/j.neuron.2011.09.006 PMC3222858

[R36] Qin P., Duncan N., Northoff G. (2013). Why and how is the self-related to the brain midline regions? *Frontiers in Human Neuroscience*, 7, 909. 10.3389/fnhum.2013.00909 PMC3872301 24399961

[R37] Qin P., Grimm S., Duncan N.W., et al. (2016). Spontaneous activity in default-mode network predicts ascription of self-relatedness to stimuli. *Social Cognitive and Affective Neuroscience*, 11(4), 693–702. 26796968 10.1093/scan/nsw008 PMC4814798

[R38] Rieger S., Göllner R., Trautwein U., Roberts B.W. (2016). Low self-esteem prospectively predicts depression in the transition to young adulthood: a replication of Orth, Robins, and Roberts (2008). *Journal of Personality and Social Psychology*, 110, 16–22. 10.1037/pspp0000037 25915130

[R39] Scalabrini A., Vai B., Poletti S., et al. (2020). All roads lead to the default-mode network—global source of DMN abnormalities in major depressive disorder. *Neuropsychopharmacology*, 45(12), 2058–69. 32740651 10.1038/s41386-020-0785-x PMC7547732

[R40] Sheline Y.I., Barch D.M., Price J.L., et al. (2009). The default mode network and self-referential processes in depression. *Proceedings of the National Academy of Sciences of the United States of America*, 106, 1942–7. 19171889 10.1073/pnas.0812686106 PMC2631078

[R41] Singelis T.M. (1994). The measurement of independent and interdependent self-construals. *Personality and Social Psychology Bulletin*, 20, 580–91.

[R42] Spreng R.N., Grady C.L. (2010). Patterns of brain activity supporting autobiographical memory, prospection, and theory of mind, and their relationship to the default mode network. *Journal of Cognitive Neuroscience*, 22, 1112–23. 19580387 10.1162/jocn.2009.21282

[R43] Teo A.R., Choi H., Valenstein M. (2013). Social relationships and depression: ten-year follow-up from a nationally representative study. *PLoS One*, 8(4), e62396. 10.1371/journal.pone.0062396 PMC3640036 23646128

[R44] Tozzi L., Goldstein-Piekarski A.N., Korgaonkar M.S., Williams L.M. (2019). Connectivity of the cognitive control network during response inhibition as a predictive and response biomarker in major depression: evidence from a randomized clinical trial. *Biological Psychiatry*, 87, 462–72. 31601424 10.1016/j.biopsych.2019.08.005 PMC8628639

[R45] Trivedi M.H., McGrath P.J., Fava M., et al. (2016). Establishing moderators and biosignatures of antidepressant response in clinical care (EMBARC): rationale and design. *Journal of Psychiatric Research*, 78, 11–23. 27038550 10.1016/j.jpsychires.2016.03.001 PMC6100771

[R46] van der Velden A.M., Kuyken W., Wattar U., et al. (2015). A systematic review of mechanisms of change in mindfulness-based cognitive therapy in the treatment of recurrent major depressive disorder. *Clinical Psychology Review*, 37, 26–39. 25748559 10.1016/j.cpr.2015.02.001

[R47] van Grieken R.A., Kirkenier A.C., Koeter M.W., Nabitz U.W., Schene A.H. (2015). Patients’ perspective on self‐management in the recovery from depression. *Health Expectations*, 18, 1339–48. 23910797 10.1111/hex.12112 PMC5060814

[R48] Wagar B.M., Cohen D. (2003). Culture, memory, and the self: an analysis of the personal and collective self in long-term memory. *Journal of Experimental Social Psychology*, 39(5), 468–75.

[R49] Wang L., Hermens D.F., Hickie I.B., Lagopoulos J. (2012). A systematic review of resting-state functional-MRI studies in major depression. *Journal of Affective Disorders*, 142, 6–12. 22858266 10.1016/j.jad.2012.04.013

[R50] Watkins E., Teasdale J.D. (2004). Adaptive and maladaptive self-focus in depression. *Journal of Affective Disorders*, 82, 1–8. 15465571 10.1016/j.jad.2003.10.006

[R51] Yan C., Zang Y. (2010). DPARSF: a MATLAB toolbox for ‘pipeline’ data analysis of resting-state fMRI. *Frontiers in Systems Neuroscience*, 4, 13. 10.3389/fnsys.2010.00013 PMC2889691 20577591

[R52] Yeh C., Hwang M. (2000). Interdependence in ethnic identity and self: implications for theory and practice. *Journal of Counseling and Development*, 78, 420–9.

[R53] Yeo B.T., Krienen F.M., Sepulcre J., et al. (2011). The organization of the human cerebral cortex estimated by intrinsic functional connectivity. *Journal of Neurophysiology*, 106, 1125–65. 21653723 10.1152/jn.00338.2011 PMC3174820

[R54] Zhang J., Cheng W., Liu Z., et al. (2016). Neural, electrophysiological and anatomical basis of brain-network variability and its characteristic changes in mental disorders. *Brain*, 139, 2307–21. 27421791 10.1093/brain/aww143

