# Supplementary material for: Self-dependent neural variability predicts recovery from depressive symptoms
Source: Soc Cogn Affect Neurosci. 2021 May 15;16(9):962–71. doi: 10.1093/scan/nsab050 (PMC8421703; doi:10.1093/scan/nsab050)
Supplement: nsab050_Supp [file nsab050_supp.zip › Supplementary materials.docx]

**Supplementary materials**

**Self-dependent neural variability predicts** **recovery from depressive symptoms**

**Leyi Fan^1^, Qin Duan^1^, Siyang Luo^1^**

**^1^Department of Psychology,**

**Guangdong Key** **Laboratory of Social Cognitive Neuroscience and Mental Health,**

**Guangdong Provincial Key Laboratory of Brain Function and Disease,**

**Sun Yat-sen University**

**Guangzhou 510006, China**

**Running head: Self-Dependent Neural Variability and Depression**

**Address correspondence to:**

**Prof. Siyang Luo, Ph.D.**

**Department of Psychology**

**Sun Yat-sen University**

**Guangzhou 510006, China**

**Phone: 86 020-3933-6573**

**Email: Luosy6@mail.sysu.edu.cn/Ljc520ida@163.com**

1. **Materials and Methods**

**1.1 Participants**

A total of 239 individuals participated in depressive symptom screening. These participants were supposed to complete the Beck Depression Inventory-II (BDI-II; Beck et al., 1996). The BDI included 21 sets of statements with 4 statements in each set. Sixty individuals (42 males and 18 females; age = 20.11 ± 2.33) participated in the fMRI studies. Among the sixty individuals, the 28 participants with the BDI score higher than 14 (range: 14-28, mean ± SD=19.21 ± 2.19) were the high-depression group, and the other 32 participants with the BDI score lower than 4 (range: 0-4, mean ± SD=2.25 ± 1.44) were the low-depression group. The participants completed the BDI again 3 months after the first screening. Recovery from depressive symptoms was assessed by subtracting the BDI score at the second screening from the BDI score at the first screening. Higher scores indicated that the participants had become less depressive. None of the participants took psychotropic medicine or treatment between the two screenings. Written informed consent was obtained from all participants before starting the experiments. All studies were approved by the ethics committee of the Department of Psychology at Sun Yat-sen University.

**1.2 Procedure and stimuli**

During the fMRI scanning, the participants completed a 5-min resting state and a self-reflection task (Figure 1). During the task, the participants were supposed to judge whether a given item described themselves (self-related conditions) or a public figure (other-related conditions; i.e. Liu Xiang, a famous athlete). The items were divided into 3 categories: mental attributes (i.e. personality characteristics), physical attributes (i.e. physical appearances) and social attributes (i.e. social identities). There were 48 items in each category. A font judgment (bold vs. light-faced) was used as the control condition. There were 6 scans with 7 blocks in each scan. Each block presented a type of judgment. The blocks presented in a random order and with a 10-s interval. Each item presented for 2 s and followed by a 1-s fixation.

After the scanning, participants completed the SCS (Singelis, 1994). The SCS was divided into two dimensions: the interdependent subscale and the independent subscale. Each subscale included 12 items that were rated on a 7-point Likert scale (1 = strongly disagree, 7 = strongly agree). Interdependence was assessed by subtracting the mean score on the independent subscale from the mean score on the interdependent subscale. Higher scores indicated more interdependent on social contexts and others.

**1.3 Imaging acquisition and preprocessing**

We used a GE Signa MR750 3.0T scanner with a standard head coil to acquire the fMRI data. The data were acquired using T2-weighted, gradient-echo, echo-planar imaging (EPI) sequences with the following parameters: repetition time (TR) = 2000 ms, echo time (TE) = 30 ms, flip angle (FA) = 90°, field of view (FOV) = 240 × 240 mm, matrix = 64 × 64 × 32, and spatial resolution = 3.75 × 3.75 × 5 mm^3^. During the resting-state scanning, the participants were supposed to keep their eyes open.

A standard preprocessing procedure was performed on the fMRI data using the Data Processing Assistant for Resting-State fMRI (DPARSF) toolbox (Yan & Zang, 2010). The data of the first five volumes were removed. The remaining data underwent slice timing and realignment to correct the time delay of scans and head motion. None of the participants was excluded during realignment because their maximum head motion were all within the criterion of 3.0 mm and 3.0 degree. The corrected data were registered to the Montreal Neurological Institute (MNI) space with the EPI template. The normalized data were Gaussian smoothed (full-width at half-maximum (FWHM) = 4 mm), detrended, and bandpass filtered (0.01-0.08 Hz). Nuisance covariates were removed by multiple regression, including rigid-body 6 head motion parameters and the mean time courses of the white matter and cerebrospinal fluid. In addition, we used different nuisance covariates removal strategies (removal of the global signal vs. no removal) to test the robustness of the prediction effects across nuisance covariates removal strategies (see supplementary results).

A 264-node atlas (Power et al., 2011) was used to define nodes and networks. The atlas included the cerebral cortex, subcortical structures and cerebellum. The nodes were 264 spheres (diameter = 10 mm) and divided into 14 networks: the auditory network, cerebellar network, cingulo-opercular task control network, default mode network, dorsal attention network, frontoparietal task control network, hand sensory-somatomotor network, memory retrieval network, mouth sensory-somatomotor network, salience network, subcortical network, uncertain network, ventral attention network, and visual network.

**1.4 Estimation of neural variability**

We used the method proposed by Zhang et al. (2016) to estimate neural variability. The neural variability of each node was defined as the temporal variability of functional connectivity in the node.

**2.4.1 Estimation of general neural variability**

We used the fMRI data during the resting state to estimate general neural variability. Time series were extracted and split into 9 nonoverlapping windows with a length of 30 s. In space, within each window, we calculated the functional connectivity matrix using Pearson correlation analysis. Each row (or each column) of the matrix was the functional connectivity between a specific node and the remaining nodes across the whole brain. In time, for each node, we compared the functional connectivity matrices across different windows using Pearson correlation analysis. General neural variability of the node was calculated by subtracting the mean of correlation coefficients from one. The formula was as follows:

$$V_{k}=1-\bar{corrcoef\left( F_{i.k,}F_{j.k} \right)},i,j=1,2,3,\ldots,9,i\neq j,$$

where k indexed the node, and i and j indexed windows.

**2.4.2 Estimation of the difference in neural variability between the self-related conditions and other-related conditions**

We used the fMRI data during the self-reflection task to estimate the difference in neural variability between the self-related conditions and other-related conditions. The neural variability of each condition was calculated by comparing regional functional connectivity matrices across different scans of the same condition. The procedure was similar to the estimation of general neural variability. The difference in neural variability was calculated by subtracting the mean neural variability in the 3 other-related conditions from the mean neural variability in the 3 self-related conditions.

**1.5 Prediction analysis**

We used the leave-one-out cross-validation method to study whether self-dependent neural variability could predict recovery from depressive symptoms of a novel individual. The prediction analysis was divided into the following stages: feature selection, model establishment, prediction and model evaluation. The stages from feature selection to prediction formed an iteration. In each iteration, one participant was excluded as the test set, and the remaining participants were the training set. The training set established the models, and the test set evaluated the models. Because each of 60 participants was excluded once, there were 60 iterations.

**1.5.1 Self-construal-dependent neural variability predictive models**

Before the prediction analysis, the dot product of normalized general neural variability and normalized interdependence was defined as the interaction between general neural variability and interdependence. We used the interaction between general neural variability and interdependence to study whether self-construal moderated the prediction effects of general neural variability on recovery from depressive symptoms for an individual.

In the feature-selection stage, based on the training set, we performed Pearson correlation analysis between the interactions of each node and recovery (feature-selection threshold = 0.05). The nodes whose interactions were significantly positively correlated with recovery were selected as positive features, and the significantly negatively correlated nodes were negative features. In addition, we used different feature-selection thresholds (thresholds = 0.05, 0.01) to test the robustness of the prediction effects across feature-selection thresholds (see supplementary results).

In the model-establishment stage, positive features or the opposite number of negative features were averaged, resulting in the positive feature interaction strength or the negative feature interaction strength, and they were averaged together, resulting in the total feature interaction strength. Simple linear regression were used to construct the relationships between the three feature interaction strengths and recovery, resulting in three models: the total model, positive model and negative model.

In the prediction stage, based on the test set, the same features as the training set were extracted, and the three interaction strengths were substituted in the corresponding model, resulting in the predicted recovery for the participant in each of the three models.

In the model-evaluation stage, the Pearson correlation coefficient between the predicted recovery calculated by the model and the observed recovery assessed by the scale was defined as the predictive power of the model. Only the significant positive predictive power indicated that the prediction was successful. When no feature was selected in at least one iteration, we did not perform the prediction analysis to maintain the consistency of the iteration number. The nodes that were selected as features in more than 95% of the iterations were regarded as the important nodes.

To further confirm the significance of the prediction effects, we performed permutation tests. In each permutation, we shuffled the recovery scores and reran the prediction analyses. The permutations were repeated 1000 times in the total model, positive model and negative model. Thus, we generated a null distribution of the prediction effects for each of the three models.

To test the specificity of the predictive models, we tested whether general neural variability itself could predict recovery from depressive symptoms, whether the predictive models could predict the BDI score at the first assessment or at the second assessment. In addition, we tested whether head motion patterns during the resting state were correlated with recovery from depressive symptoms using Pearson correlation analysis. Six series of head motion patterns of each participant were estimated using the DPARSF toolbox (Yan & Zang, 2010). We then extracted the maximum of the absolute value of each series, resulting in six head motion parameters that included three translation parameters and three rotation parameters. We used the sum of all six parameters, the sum of the three translation parameters, and the sum of the three rotation parameters to conduct correlation analyses.

**1.5.2 Self-referential-related neural variability predictive models**

The procedure was similar to the prediction analyses of the interaction between general neural variability and interdependence except that the interaction was replaced by the differences in neural variability. To study whether self-related neural variability or other-related neural variability could predict recovery from depressive symptoms, we also used the differences in neural variability between the self-related conditions and the font condition or the differences in neural variability between the other-related conditions and the font condition to perform the prediction analyses. To further confirm the significance of the prediction effects, we performed permutation tests.

Moreover, for the domain-specific prediction effect, we used the differential neural variability between self-related conditions and other-related conditions in which participants made judgments about mental attributes, physical attributes or social attributes to perform the prediction analyses.

To test the specificity of the predictive models, we tested whether the predictive models could predict the BDI score at the first assessment or at the second assessment. In addition, we tested whether head motion patterns during the self-reflection task were correlated with recovery from depressive symptoms using Pearson correlation analysis.

1. **Results**

**2.1 Robustness across nuisance covariate removal strategies**

To test the robustness of the prediction effects across nuisance covariate removal strategies, we used different nuisance covariate removal strategies (no removal of the global signal vs. removal) in the imaging preprocessing.

After the removal of the global signal, the interaction between general neural variability and interdependence remained predictive of recovery from depressive symptoms in the total model (*r* = 0.29, *p* = 0.024) and in the positive model (*r* = 0.29, *p* = 0.024) while the prediction effect was not significant in the negative model (*r* = -0.01, *p* = 0.914).

The results showed that the prediction effects were robust across nuisance covariate removal strategies.

**2.2 Robustness across feature-selection thresholds**

To test the robustness of the prediction effects across feature-selection thresholds, we used different thresholds (thresholds = 0.05, 0.01) in the feature-selection stage.

In the self-construal-dependent neural variability predictive models, at the feature-selection threshold of 0.01, the interaction between general neural variability and interdependence remained predictive of recovery from depressive symptoms in the total model (*r* = 0.32, *p* = 0.013) and in the positive model (*r* = 0.32, *p* = 0.013). In the negative model, no feature was selected in at least one iteration, so the prediction analysis was not performed.

In the self-referential-related neural variability predictive models, at the feature-selection threshold of 0.01, the differences in neural variability between the self-conditions and other-conditions during the self-referential task remained predictive of recovery from depressive symptoms in the total model (*r* = 0.37, *p* = 0.003) and in the negative model (*r* = 0.37, *p* = 0.004). Moreover, the prediction effects remained significant when participants made judgements about mental attributes in the total model (*r* = 0.30, *p* = 0.019) and in the negative model (*r* = 0.30, *p* = 0.019). In the positive model, no feature was selected in at least one iteration, so the prediction analysis was not performed.

The results showed that the prediction effects were robust across feature-selection thresholds.

**2.3 Specificity of the predictive models**

**2.3.1 Self-construal-dependent neural variability predictive models**

We tested whether the interaction between general neural variability and interdependence could predict the BDI score at the first assessment or at the second assessment with the important nodes as features. The interaction within the important nodes failed to predict the BDI score at the first assessment (*r* = 0.19, *p* = 0.132) or at the second assessment (*r* = -0.40, *p* = 0.001). In addition, we tested whether head motion patterns during the resting state were correlated with recovery from depressive symptoms. The sum of all six parameters (*r* = -0.07, *p* = 0.584), the sum of the three translation parameters (*r* = -0.18, *p* = 0.170) and the sum of the three rotation parameters (*r* = 0.03, *p* = 0.818) were not significantly correlated with recovery from depressive symptoms. The results illustrated the specific effect of self-construal-dependent neural variability in predicting recovery from depressive symptoms.

**2.3.2 Self-referential-related neural variability predictive models**

We then tested whether the differences in neural variability between the self-related conditions and other-related conditions could predict the BDI score at the first assessment or at the second assessment with the important nodes as features. The differences in neural variability within the important nodes failed to predict the BDI score at the first assessment (*r* = -0.07, *p* = 0.587) or at the second assessment (*r* = -0.10, *p* = 0.449). The prediction effects were also not significant in the mental condition (first assessment: *r* = 0.00, *p* = 0.996; second assessment: *r* = -0.21, *p* = 0.100). In addition, we tested whether head motion patterns during the self-reflection task were correlated with recovery from depressive symptoms using Pearson correlation analyses. The sum of all six parameters (*r* = -0.01, *p* = 0.926), the sum of the three translation parameters (*r* = 0.02, *p* = 0.859) and the sum of the three rotation parameters (*r* = -0.04, *p* = 0.738) were not significantly correlated with recovery from depressive symptoms. The results illustrated the specificity of the self-referential–related neural variability predictive models.

**References**

Beck, A. T., Steer, R. A., & Brown, G. K. (1996). *Manual for the Beck Depression Inventory-II*. San Antonio: The Psychological Corporation.

Power, J. D., Cohen, A. L., Nelson, S. M., Wig, G. S., Barnes, K. A., Church, J. A. et al. (2011). Functional network organization of the human brain. *Neuron*, *72*, 665-678.

Singelis, T. M. (1994). The measurement of independent and interdependent self-construals. *Personality and Social Psychology Bulletin*, *20*, 580-591.

Yan, C., & Zang, Y. (2010). DPARSF: a MATLAB toolbox for “pipeline” data analysis of resting-state fMRI. *Frontiers in Systems Neuroscience*, *4*, 13.

Zhang, J., Cheng, W., Liu, Z., Zhang, K., Lei, X., Yao, Y. et al. (2016). Neural, electrophysiological and anatomical basis of brain-network variability and its characteristic changes in mental disorders. *Brain*, *139*, 2307-2321.
